# Metabolic Signature-Based Subtypes May Pave Novel Ways for Low-Grade Glioma Prognosis and Therapy

**DOI:** 10.3389/fcell.2021.755776

**Published:** 2021-11-23

**Authors:** Ganglei Li, Zhanxiong Wu, Jun Gu, Yu Zhu, Tiesong Zhang, Feng Wang, Kaiyuan Huang, Chenjie Gu, Kangli Xu, Renya Zhan, Jian Shen

**Affiliations:** ^1^ Department of Neurosurgery, The First Affiliated Hospital, College of Medicine, Zhejiang University, Hangzhou, China; ^2^ School of Electronic Information, Hangzhou Dianzi University, Hangzhou, China

**Keywords:** low-grade glioma, metabolic signature, subtypes, prognosis, immune characteristics

## Abstract

Metabolic signatures are frequently observed in cancer and are starting to be recognized as important regulators for tumor progression and therapy. Because metabolism genes are involved in tumor initiation and progression, little is known about the metabolic genomic profiles in low-grade glioma (LGG). Here, we applied bioinformatics analysis to determine the metabolic characteristics of patients with LGG from the Cancer Genome Atlas (TCGA) and the Chinese Glioma Genome Atlas (CGGA). We also performed the ConsensusClusterPlus, the CIBERSORT algorithm, the Estimate software, the R package “GSVA,” and TIDE to comprehensively describe and compare the characteristic difference between three metabolic subtypes. The R package WGCNA helped us to identify co-expression modules with associated metabolic subtypes. We found that LGG patients were classified into three subtypes based on 113 metabolic characteristics. MC1 patients had poor prognoses and MC3 patients obtained longer survival times. The different metabolic subtypes had different metabolic and immune characteristics, and may have different response patterns to immunotherapy. Based on the metabolic subtype, different patterns were exhibited that reflected the characteristics of each subtype. We also identified eight potential genetic markers associated with the characteristic index of metabolic subtypes. In conclusion, a comprehensive understanding of metabolism associated characteristics and classifications may improve clinical outcomes for LGG.

## Introduction

Low-grade glioma (LGG) is the most common slow-growing brain cancer in central nervous system neoplasms (De Andrade Costa et al., 2021). Diffusely infiltrating LGGs include astrocytomas, oligodendrogliomas, and mixed oligoastrocytomas (WHO grade 2) (Louis et al., 2016). LGGs are typically nonmalignant and slow-growing, and account for 6.4% of all adult primary CNS tumors. LGGs are characterized as indolent tumors, with survival rates that range from 1 to 15 years (Gargini et al., 2020). The long-term survival of LGG mainly depends on the resection extension, molecular subtyping such as isocitrate dehydrogenase (IDH) 1 and 2 mutations (Huang et al., 2020), and 1p19q codeletion (Merchant et al., 2009; Gargini et al., 2020). LGGs exhibit widespread genetic and phenotypic heterogeneity, which is characterized by a mutation in the IDH enzyme (Binder et al., 2019). Most LGGs inevitably progress to higher-grade tumors, and about 50–75% LGG patients often evolves to pathological progression and deterioration. Hence, an intensive exploration of the regulation mechanism in LGG initiation and progression is vital for biomarker identification and determination of therapeutic targets.

Aberrant cellular metabolism alters the metabolic and immune microenvironments, and has emerged as a therapeutic target in cancer diagnosis and therapy. The evidence indicates that metabolism-associated genes may contribute to progression by altering tumor metabolism and behavior or impacting the tumor microenvironment (Chen et al., 2019). Metabolism-associated genes play key roles in cancerous cells, and cancerous cell-metabolism reprogramming is considered the new direction for future cancer research (Hanahan and Weinberg, 2011). Studies have demonstrated that metabolic alterations may promote tumor cell proliferation and migration (Zhu et al., 2020). Therefore, metabolism-associated genes may be a fruitful focus for identifying the genomic profiles and inner regulation mechanism of LGG.

In the current study, we applied bioinformatics analysis based on the Cancer Genome Atlas (TCGA) and the Chinese Glioma Genome Atlas (CGGA). We performed the ConsensusClusterPlus to identify metabolic subtypes, the CIBERSORT algorithm to calculate relative immune abundance, the Estimate software application to evaluate immune infiltration and the R package “GSVA” for enrichment analysis. TIDE and the R package WGCNA were applied to evaluate potential clinical effects in immunotherapy and identify co-expression modules with associated metabolic subtypes. In the end, we also identified eight potential biomarkers reflecting metabolic subtype characteristics which have potential become novel therapeutic targets for LGG therapies.

## Materials and Methods

### Data Collection

We downloaded LGG patients’ gene transcriptome profiles and corresponding RNA-seq data from TCGA database (https://portal.gdc.cancer.gov/) and the Genomic Data Commons (GDC) tool. 509 LGG samples logged in TCGA were available for analysis. Additionally, mRNA-seq 693 (batch 1) and mRNA_seq325 (batch 1) datasets were downloaded from CGGA (http://www.cgcg.org.cn/). Next, these two RNA-seq datasets were merged into one metadata set; 408 samples were ultimately included in this study.

### Metabolism-Relevant Gene Selection

Previous studies by other researchers screened a set of metabolic genes relevant to malignant tumor activity and stemness properties required for tumorigenesis (Possemato et al., 2011). A total of 2,752 metabolism-relevant genes which encoded metabolic enzymes and transporters were selected.

### Genomic Data Pre-treatment

Poor-quality samples were excluded before data preprocessing. Samples without clinical data and with more than 50% missing were removed. In the end, we obtained an expression profile dataset containing 20,485 genes profiles for subsequent analysis.

### Metabolic Subgroup Classification

ConsensusClusterPlus implements the consensus clustering (CC) method, which facilitates more quantitative stability evidence in unsupervised class discovery (Wilkerson and Hayes, 2010). We used the Normalized Enrichment Score (NES) to measure the gene-sets enrichment (Wilkerson and Hayes, 2010; Yang et al., 2020), and obtained 113 normalized enrichment scores of metabolism-relevant gene signatures. Each of the 113 metabolism-related signatures had a class of gene sets and contained multiple genes. We adopted the “PAM” algorithm along with “Canberra” as a measure of distance, and performed 500 bootstraps, each involving 80% of the patients in the training set. The clustering number was set as 2–10, and the consistency matrix and consistency cumulative distribution function were calculated to determine the best classification.

### Tumor Immune Infiltration

To infer the relative abundance of 22 types of tumor-infiltrating immune cells and non-immune cells in the tumor microenvironment, we applied the CIBERSORT algorithm via the “CIBERSORT” R package (CIBERSORT R script v1.03; http://cibersort.stanford.edu/). We adopted the ESTIMATE package to determine the presence of infiltrating immune cells (Yu et al., 2019), using the ImmuneSignature gene set based on LGG RNA-seq data (Jusakul et al., 2017).

### Gene Set Variation Analysis and Functional Annotation

To further explore the differences between different clusters in biological processes, we performed the gene set variation analysis (GSVA) with the R package “GSVA” to estimate pathway enrichment for different clusters. In total, 113 metabolism-associated pathways were included in GSVA analysis genesets. R package clusterProfiler (https://guangchuangyu.github.io/software/clusterProfiler) was used to process the GSEA analysis.

### Metabolic Subtype Characteristic Score Construction

Considering that different metabolic characteristics existed in different metabolic subtypes, we applied the principal components analysis (PCA). And we established subtype classification scores better to quantify metabolic characteristics of patients in different sample cohorts. Specifically, we used 113 metabolic characteristics for PCA analysis, and the first two-component scores. Then, we calculated the metabolic subtype characteristic score of each sample and calculate the formula MRGs-score = ∑(PC1 I + PC2 I), where I represented metabolic characteristics.

## Results

### Metabolism-Associated Gene Identification and Classification

**FIGURE 1:**
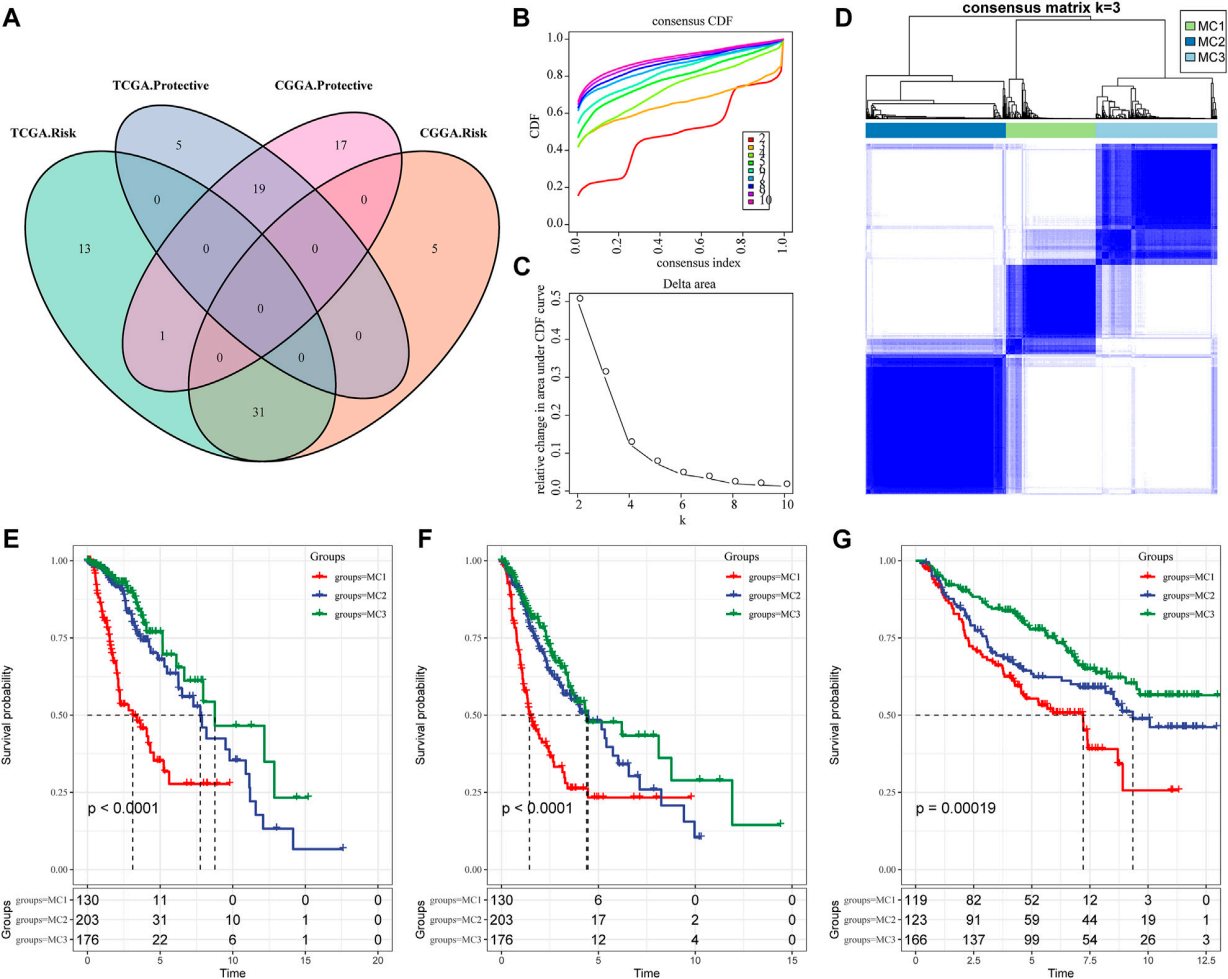
Metabolic subtypes in LGGA. **(A)** The intersection of prognostic metabolic signatures between TCGA and CGGA. **(B)** CDF curve of the TCGA cohort. **(C)** CDF delta area curve. The horizontal axis represents the category number k and the vertical axis represents the relative change in area under the CDF curve. **(D)** Sample clustering heatmap, k = 3. **(E)** Overall survival curve of three metabolic subtypes in the TCGA cohort. **(F)** PFS curve of metabolic subtypes in TCGA cohort. **(G)** OS curves of three subtypes in the CGGA cohort.

To identify prognosis-related metabolic signatures, we calculated enrichment score and prognosis features of 113 metabolic genes from LGG patients in the TCGA and CGGA cohorts. The results indicated that there were 69 prognosis-related metabolic signatures in the TCGA database, and 73 prognosis-associated metabolic genes (Figure 1A). Furthermore, we adopted ConsensusClusterPlus to explore reasonable classifications according to the characteristics of the 509 LGG sample, and classified these similar characteristics genes into one category. The corresponding cumulative distribution function (CDF) curve and the delta area plot indicated that the optimal choice was K = 3 (Figures 1B,C). Finally, we obtained three different metabolic clusters (MC) which were characterized by consistent metabolic subtype-related specificities (Figure 1D). We further investigated the prognosis linked to these three clusters. Results for overall survival (OS) indicated that MC1 predicted the shortest survival time compared with MC2 and MC3 in the TCGA dataset (Figure 1E). The progression-free survival (PFS) outcomes showed that the MC1 subgroup had the poorest survival rates in both the TCGA cohort and the CGGA cohort (Figures 1F,G).

### Distinct Clinical Signatures and Outcomes for the Three Metabolic Clusters of Low-Grade Glioma

To clarify the clinical signatures between the three clusters, the results demonstrated that there was no significant difference in occurrence of any signature based on age or gender (Supplementary Figures S1A,B). There was a remarkable variation in IDH mutation in the TCGA database, and IDH mutation in the MC2 and MC3 subgroups was significantly higher than that in the MC1 subgroup (Supplementary Figure S1C). The probabilistic infiltration weighted gradient maps also demonstrated that the chromosome 1p and 19q (1p/19q) codeletion status was significantly higher compared with MC2 and MC1 in the TCGA dataset (Supplementary Figure S1D). The methylguanine-DNA methyl-transferase (MGMT) expression in MC1 was obviously decreased compared with the MC2 and MC3 subgroups (Supplementary Figure S1E). At the same time, we explored these features in the CGGA database, and noticed that consistent with the prior observations, the three clusters did not differ significantly based on age or gender (Supplementary Figures S1F,G). IDH mutation and 1p19q co-deletion were significantly higher in MC2 and MC3 compared with MC1 (Supplementary Figures S1H,I). MGMT expression was not significantly different in the three subgroups (Supplementary Figure S1J).

### Metabolic Characteristics of the Three Clusters

To thoroughly investigate the metabolic characteristics, we applied differential expression analysis to identify subtype-specific metabolic characteristics based on the GSVA scoring system. According to the results, 47 specific metabolic characteristics were present in MC1, 6 in MC2, and 39 in MC3, as visualized by the heatmap (Supplementary Figure S2). These genes were applied to further distinguish between metabolic subgroups.

### Distribution of Mutation Characteristics Between the Three Metabolic Subtypes

**FIGURE 2:**
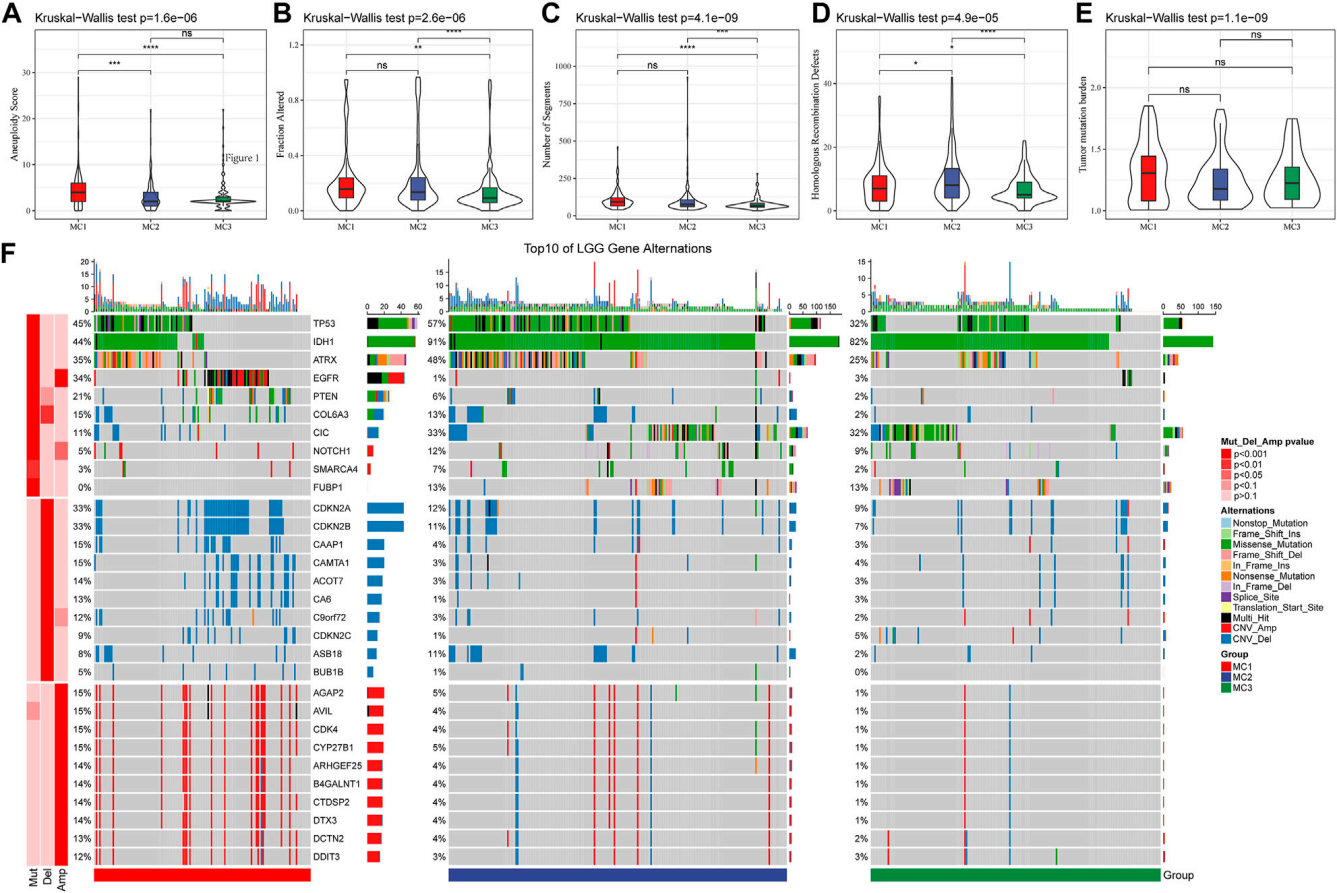
The genomic alteration of the three clusters in the TCGA cohort. **(A–E)** Comparisons of aneuploidy score, fraction altered, number of segments, tumor mutation burden and homologous recombination defects in the TCGA cohort. **(F)** The somatic mutations and copy number mutations of the three clusters.

We also analyzed the differences in genomic changes among these three metabolic subtypes in the TCGA cohort. The MC3 subtype exhibited a lower aneuploidy score (Figure 2A), fraction altered (Figure 2B), number of segments (Figure 2C), and number of homologous recombination defects (Figure 2D). Tumor mutation burden was similar in all three subgroups (Figure 2E). In addition, we analyzed the correlation between gene mutations and metabolic subtypes, and found metabolic subtypes. There was a significant correlation with gene mutation. TP53, IDH1, ATRX, and EGFR genes showed extensive somatic mutations in LGG, among which the IDH1 gene had a higher mutation frequency in MC2 and MC3 subtypes, and patients with tumors with IDH1/2 mutations had favorable prognoses. The mutation frequency of the ATRX gene in the MC2 subtype was the highest, followed by MC1. EGFR had a higher mutation frequency in the MC1 subtype, but a lower frequency in both MC2 and MC3 subtypes. In terms of copy number variation, the MC1 subtype had a wide range of copy number amplification and deletion frequencies (Figure 2F).

### Heterogeneity Analysis of Metabolic Subtypes

To comprehensively investigate the tumor heterogeneity among different metabolic subtypes, we obtained genomic characteristics including tumor purity, ploidy, amplification, and intertumoral heterogeneity from known studies (Thorsson et al., 2018). They indicated that the purity, ploidy, and intratumor Heterogeneity of MC1 and MC3 were significantly lower than those of MC2 (Supplementary Figures S3A–C). MC1 showed the highest proliferation score, followed by MC2 and MC3 (Supplementary Figure S3D). Studies have demonstrated that LGG patients with higher mRNAsi indices have a better prognosis than those with low mRNAsi indices (Tan et al., 2021) (Supplementary Figure S3E). We obtained and analyzed mRNAsi differences in LGG patients from our data, and found that the mRNAsi indices of MC2 and MC3 were significantly higher than that of MC1. We also analyzed epigenetic regulation based on index (EREGmRNAsi) differences among different metabolic subtypes, and found that there was no significant difference between EREGmRNAsi subtypes (Supplementary Figure S3F).

### Comparison of Metabolic Subtypes and Immune Cell Infiltration

**FIGURE 3:**
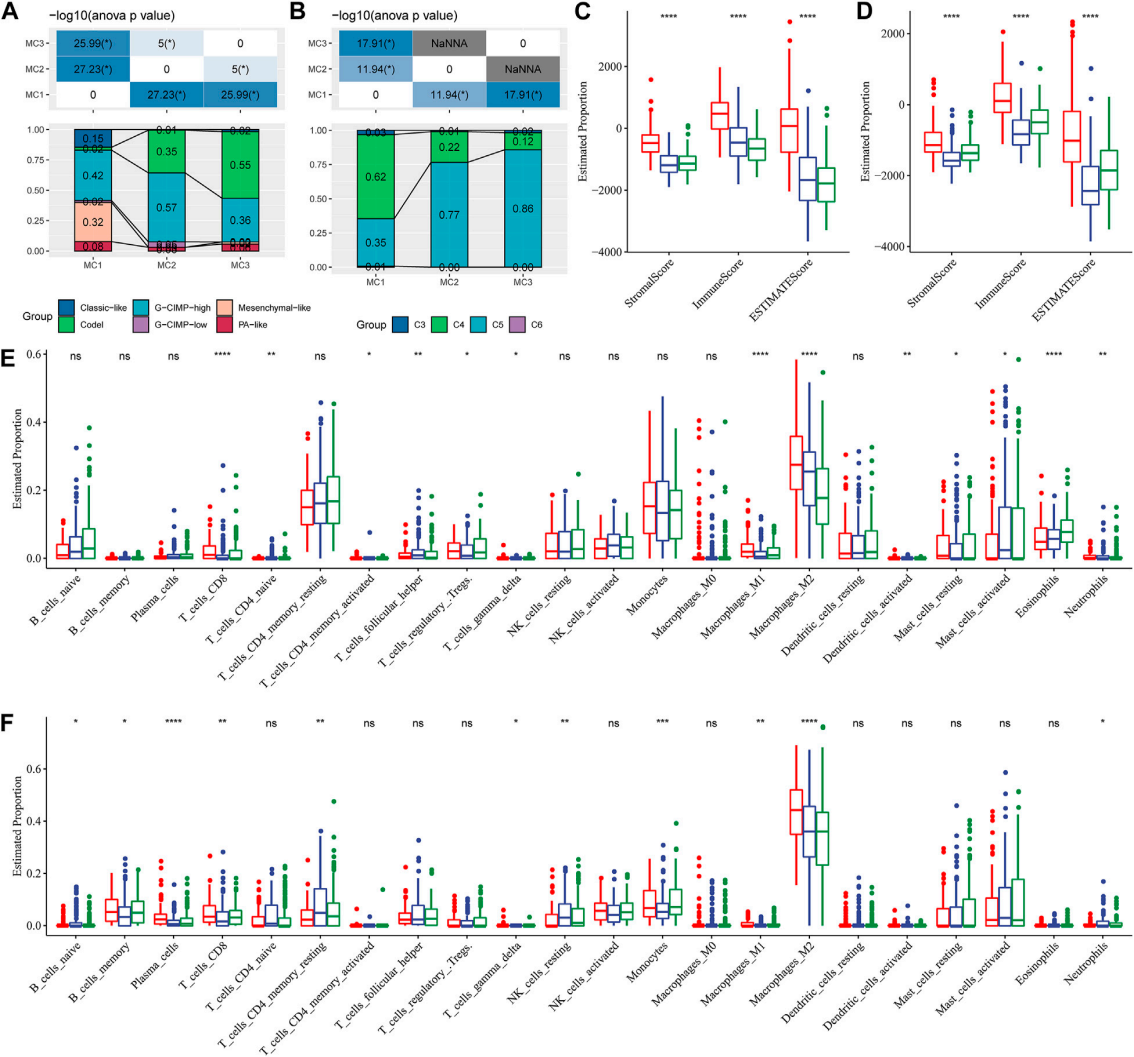
Comparison of differences in classical immune cell typing and immune cell composition analysis. **(A)** Comparative analysis of the metabolic molecular subtypes in TCGA and the reported six classical subtypes. **(B)** Comparative analysis of the metabolic molecular subtypes in TCGA and the previous six pan-cancer immune molecular subtypes. **(C)** Immune cell composition and proportion in the TCGA cohort given by ESTIMATE software. **(D)** Immune cell composition and proportion in the CGGA cohort given by ESTIMATE software. **(E)** Immune cell composition and proportion in the TCGA cohort given by CIBERSORT software. **(F)** Immune cell composition and proportion in the CGGA cohort given by CIBERSORT software.

To compare the differences between the three metabolic subtypes identified by us and the seven metabolic subtypes reported in previous studies (Ceccarelli et al., 2016), we determined that Codel and G-CIMP-high were higher in MC2 and MC3 subtypes. The percentages of patients with Codel and G-CIMP-high were 35 and 57%, respectively, in the MC2 subtype, and 55 and 36%, respectively, in the MC3 subtype. Patients with Codel and G-CIMP-high had a better prognosis (Figure 3A). We also compared the metabolic subtypes with the six previously reported immunomolecular subtypes (Thorsson et al., 2019), and found C3 to have the best prognosis and C4 and C6 subtypes to have the worst. The percentages of C5 subtype in MC2 and MC3 subtypes were 77 and 86% respectively, while the percentage of C4 subtype in the MC1 subtype was 62%, higher than those in MC2 and MC3 subtypes (Figure 3B). Immuno-infiltration analysis showed that MC1 had the highest immuno-microenvironment infiltration in the TCGA cohort, followed by MC2, and MC3 had the lowest immune-infiltration score (Figure 3C). Consistent findings were also observed in the CGGA cohort: MC1 subtype had the highest immune infiltration, significantly higher than that of MC2 and MC3 metabolic subtypes (Figure 3D). We then applied the CIBERSORT method to investigate the components of immune cells in each metabolic subtype. The results demonstrated that there were significant differences among different subtypes of immune cells. In the TCGA cohort, the Macrophages_M2 was significantly enriched in the MC1 subtype, and the CD8^+^ T cells, Naïve CD4^+^ T cells, activate CD4^+^ memory T cells, follicular helper T cells, Treg, gamma delta T cells, M1 macrophages, activated dendritic cells, mast cells, eosinophils, and neutrophils were differently expressed in the three clusters (Figure 3E). In the CGGA cohort, Macrophages_M2 in the MC1 subtype was significantly higher than that in MC2 and MC3, while naïve B cells, memory B cells, plasma cells, CD8^+^ T cells, resting memory CD4^+^ T cells, gamma delta T cells, resting NK cells, monocytes, M1 macrophages, and neutrophils were differently expressed in these classifications (Figure 3F). In addition, we also applied the xCell, EPIC, and MCP-counter to comprehensively evaluate the tumor-infiltrating immune cell score in the TCGA database and CGGA dataset. And results demonstrated that the MC1 subtype had the highest immune infiltration, while the MC2 and MC3 metabolic subtypes were significantly lower than the MC1 subtype. In addition, most immune cells had significant differences between subtypes in the TCGA database and CGGA database Supplementary Figure S4. These findings suggested that our metabolism-related classification was closely related to immune cell infiltration, which may be an effective evaluation method in LGG immune evaluation.

### Metabolism Subtypes Have Predictive Value for Immunotherapy

**FIGURE 4:**
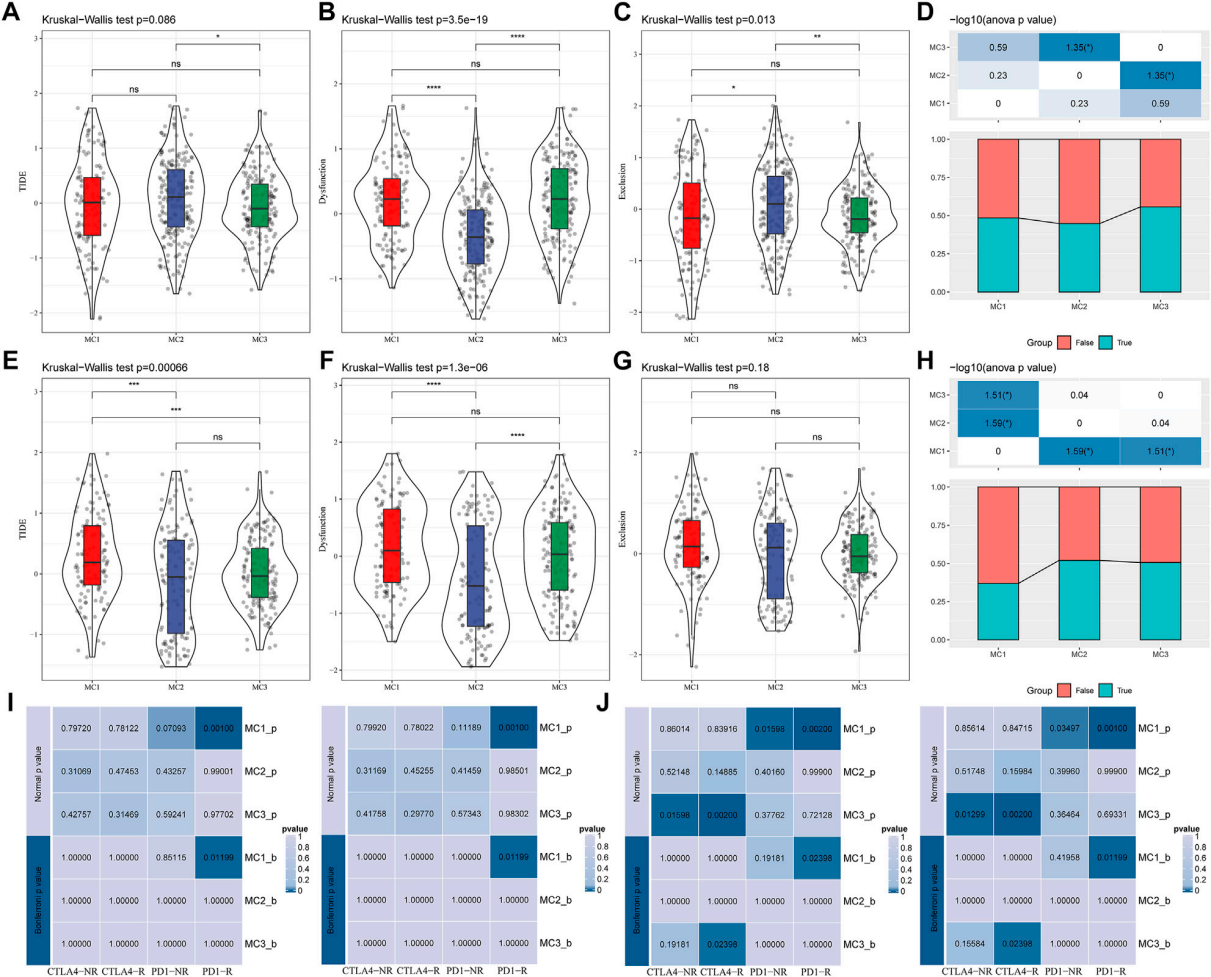
The immunotherapy response difference between the three clusters. **(A)** TIDE scores of all three metabolic subtypes in the TCGA cohort. **(B)** T cell dysfunction scores of all three metabolic subtypes in the TCGA cohort. **(C)** T cell rejection scores of different metabolic subtypes of TCGA. **(D)** Immune response status in different metabolic subtypes of TCGA. **(E)** TIDE scores of different metabolic subtypes in the CGGA cohort. **(F)** T cell dysfunction scores of different metabolic subtypes in the CGGA cohort. **(G)** T cell rejection scores of different metabolic subtypes in the CGGA cohort. **(H)** Differences of immune response status in different metabolic subtypes in the CGGA cohort. **(I)** Different immunotherapy sensitivity in programmed cell death protein 1 inhibitor therapy in the TCGA cohort. **(J)** Different immunotherapy sensitivity in programmed cell death protein 1 inhibitor therapy in the CGGA cohort.

To evaluate the different immunotherapy and potential clinical effects of metabolism-associated subtypes, we adopted TIDE (http://tide.dfci.harvard.edu/) software and determined that MC had the highest TIDE score in the TCGA cohort, which suggested a higher possibility of MC2 immune escape and a lower possibility of benefit from immunotherapy (Figure 4A). We also compared the differences in T cell dysfunction scores and T cell rejection scores among different molecular metabolic subtypes in the TCGA cohort. The results indicated that the MC2 subtype had the lowest T cell dysfunction scores but the highest T cell rejection scores. The T cell dysfunction scores of MC1 and MC3 subtypes were higher than that of the MC2 subtype, but the T cell rejection scores were lower. However, there was no significant difference in T cell dysfunction scores and T cell rejection scores between the MC1 and MC3 subtypes (Figures 4B,C). Similar results were also observed in the CGGA cohort (Figures 4E–G). In addition, we investigated the differences in the predicted immunotherapy response for different metabolic subtypes, and the results showed significant differences in immunotherapy response status between the MC2 and MC3 subtypes. In the CGGA cohort, there was a significant difference between the MC1 subtype and the MC2 and MC3 subtypes (Figures 4D,H). Furthermore, we carried out subclass mapping and compared the immunotherapy data on the three metabolic subtypes; we found that the MC1 subtype in the TCGA and CGGA cohorts was sensitive to anti-PD1 therapy, while the MC3 subtype was sensitive to CTLA4-R (Figures 4I,J).

### Construction of the Metabolic Subtype Characteristic Score

**FIGURE 5:**
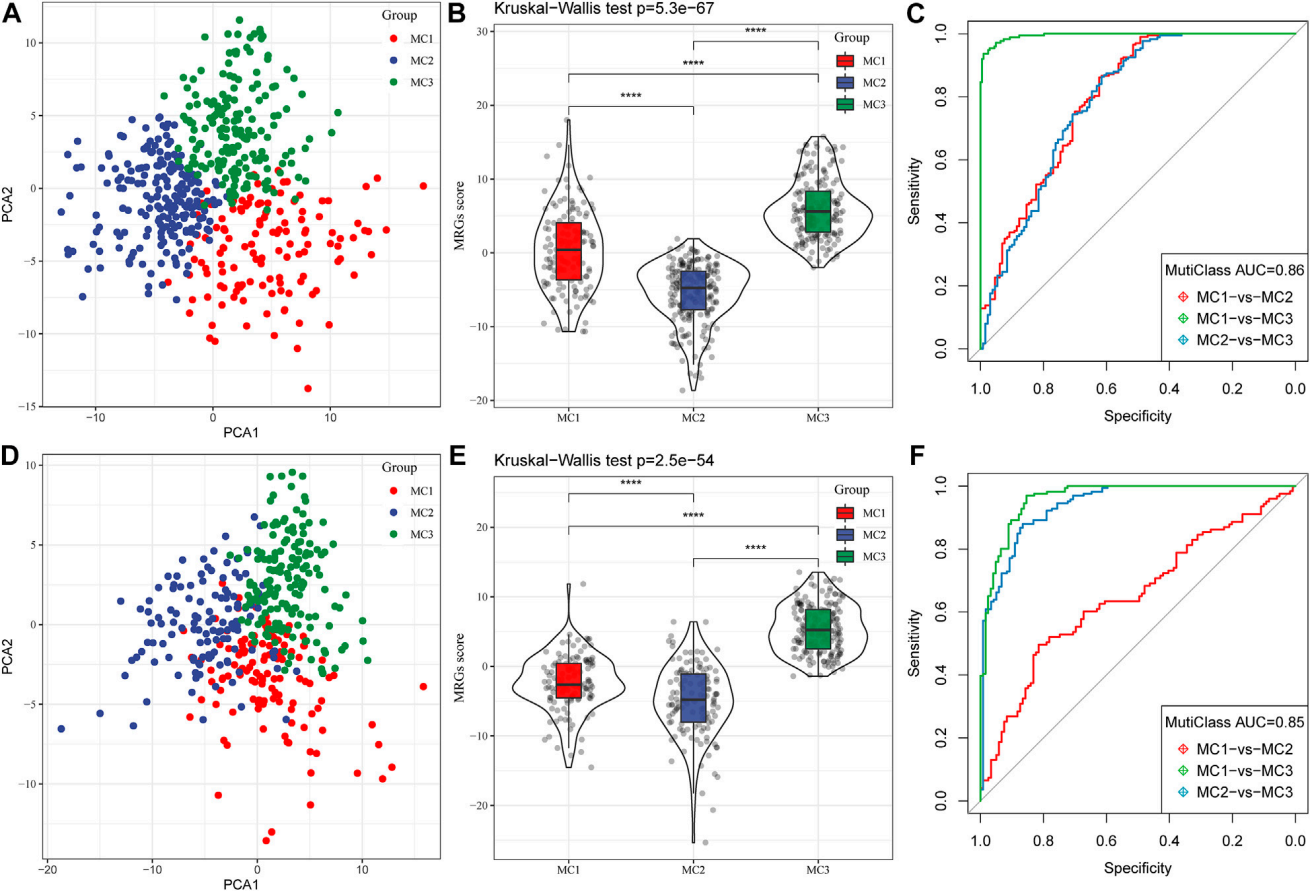
Construction of metabolic subtype characteristic score. **(A)** The relationship between two key metabolic signatures and metabolic subtypes in the TCGA cohort. **(B)** The metabolic subtype signature scores of different subtypes in the TCGA cohort. **(C)** The ROC curve for metabolic subtype signature scores in the TCGA cohort. **(D)** The relationship between two key metabolic signatures and metabolic subtypes in the CGGA cohort. **(E)** The metabolic subtype signature scores between different subtypes in the CGGA cohort. **(F)** The ROC curve for metabolic subtype signature scores in the CGGA cohort.

Because different metabolic subtypes have different metabolic characteristics, we performed PCA to construct a subtype classification feature score and thus better quantify the metabolism-related characteristics of each sample. The PCA indicated that PC1 and PC2 could successfully discriminate and classify LGG samples according to the metabolic subtypes classification features in the TCGA database (Figure 5A). The results revealed that the three metabolic subtypes had significantly different microenvironment-related genes scores (Figure 5B). The receiver-operating characteristic (ROC) curve, combining sensitivity and specificity, showed the performance of different metabolic subtype feature scores of different clusters. The multiclass area under the curve (AUC) was 0.86, which indicated that this signature score model had excellent predictive power (Figure 5C). We also observed similar features in the CGGA database (Figures 5D–F), in which the multiclass AUC was 0.85.

### Correlation Analysis Between Metabolic Subtype Signature Score and Immune Infiltration

To assess the correlation between metabolic subtype characteristics scores and immune cell characteristics, we performed Pearson’s correlation analysis, and found that metabolic subtypes signature scores had no significant correlation with immune cells, with the exception of M2 macrophages and eosinophils.

These results highlighted the fact that M2 macrophages had a remarkably strong negative correlation with metabolism-associated scores, and eosinophils had a positive correlation with these scores (Supplementary Figure S5A). The results for M2-macrophage infiltration in the CGGA cohort agreed with those from the TCGA cohort (Supplementary Figure S5B). Furthermore, to better describe the correlation analyses between metabolic subtypes, metabolic score, and immune checkpoint molecules, we adopted correlation row analysis, and we found that the metabolic subtypes, metabolic score and immune checkpoint molecules have significant correlation with each other (Supplementary Figure S6). These findings suggested that the metabolic signature had good cooperativity for not only metabolic score but also immune associated molecules.

### Co-Expressed Gene Identification

**FIGURE 6:**
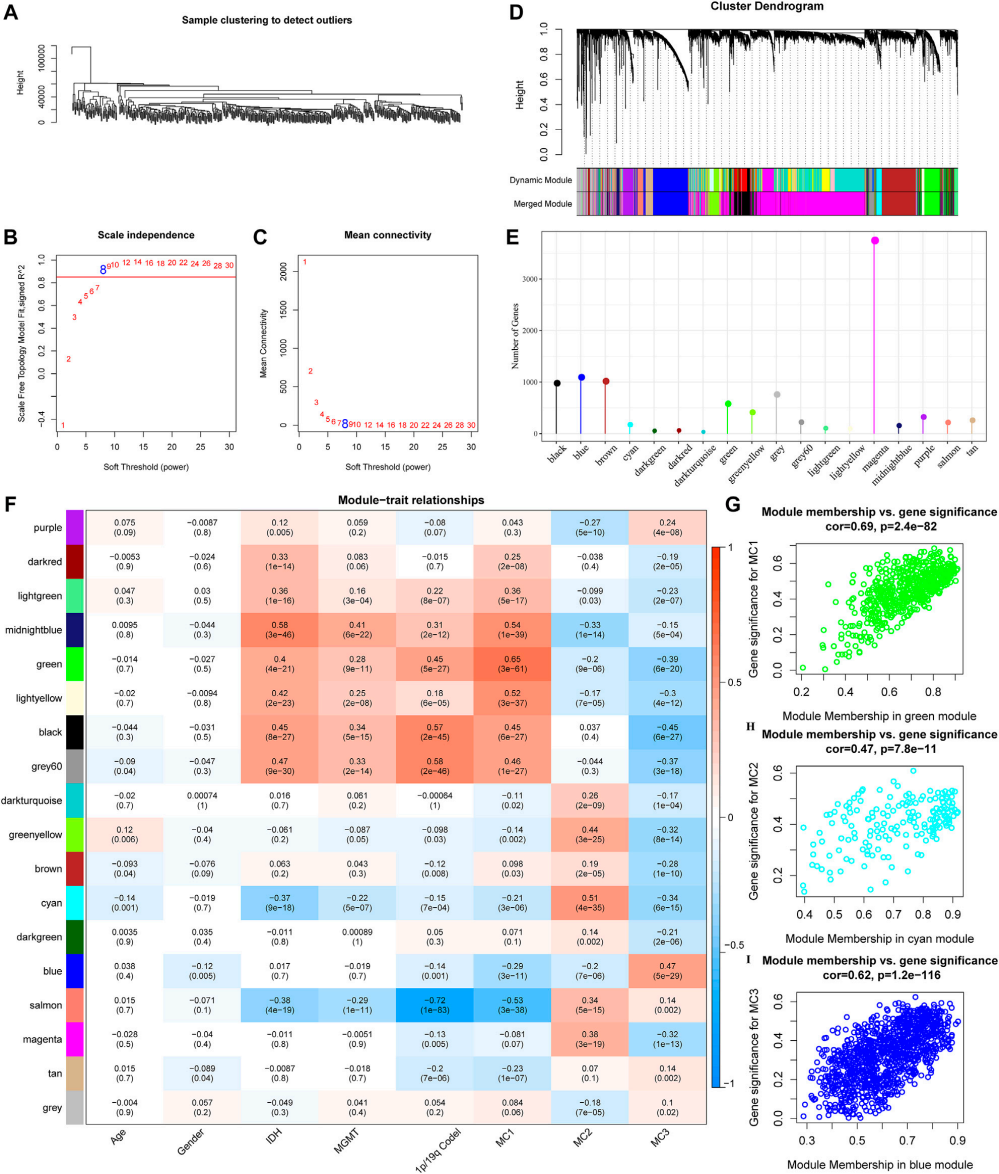
Co-expressed gene modules identification. **(A)** Clustering tree of each sample. **(B)** The scale-free fit index for various soft-thresholding powers (β). **(C)** The mean connectivity for various soft-thresholding powers. **(D)** Dendrogram of all differentially expressed genes/lncRNAs, clustered based on a dissimilarity measure. **(E)** Co-expression module gene statistical results. **(F)** Correspondence between each module and clinical information. **(G)** Scatter diagram for module membership vs gene significance for MC1 in the green module. **(H)** Scatter diagram for module membership vs gene significance for MC2 in the cyan module. **(I)** Scatter diagram for module membership vs gene significance for MC3 in the blue module.

To further identify metabolism subtypes associated with co-expressed gene models, we performed a weighted gene co-expression network analysis (WGCNA) to identify modules connected with a variety of LGG metabolism signature-based subtypes. The genes, whose MAD (median absolute difference) were more than 50%, were selected for further WGCNA analysis in the TCGA gene expression profile. The sample clusters are illustrated in Figure 6A. To ensure a scale-free network, a power of *β* = 8 (scale-free R2) was selected as the soft-thresholding parameter (Figures 6B,C). Furthermore, similar clusters were merged into new modules using the following settings: height = 0.25, deepSplit = 2, and min ModuleSize = 30. This produced 18 gene molecules (Figure 6D). The 18 gene numbers of each co-expression network are illustrated in Figure 6E. We further analyzed the correlation between each co-expressed gene module and Age, Gender, IDH1/IDH2 mutation status, MGMT methylation status, 1P/19Q CODEL status, and MC1, MC2, and MC3. The results showed that the green module had the highest correlation with MC1, the Cyan module was significantly positively correlated with MC2, and the blue module was significantly positively correlated with MC3 (Figure 6F). The Green module had a significant correlation with MC1 (Figure 6G, cor = 0.69, *p* = 2.4e−82). The Cyan module had a significant correlation with MC2 (Figure 6H, cor = 0.47, *p* = 7.8e−11). The Blue module had a significant correlation with MC3 (Figure 6I, cor = 0.62, *p* = 1.2e−116).

### Functional Enrichment Analysis of the Metabolic Co-Expression Gene Modules

**FIGURE 7:**
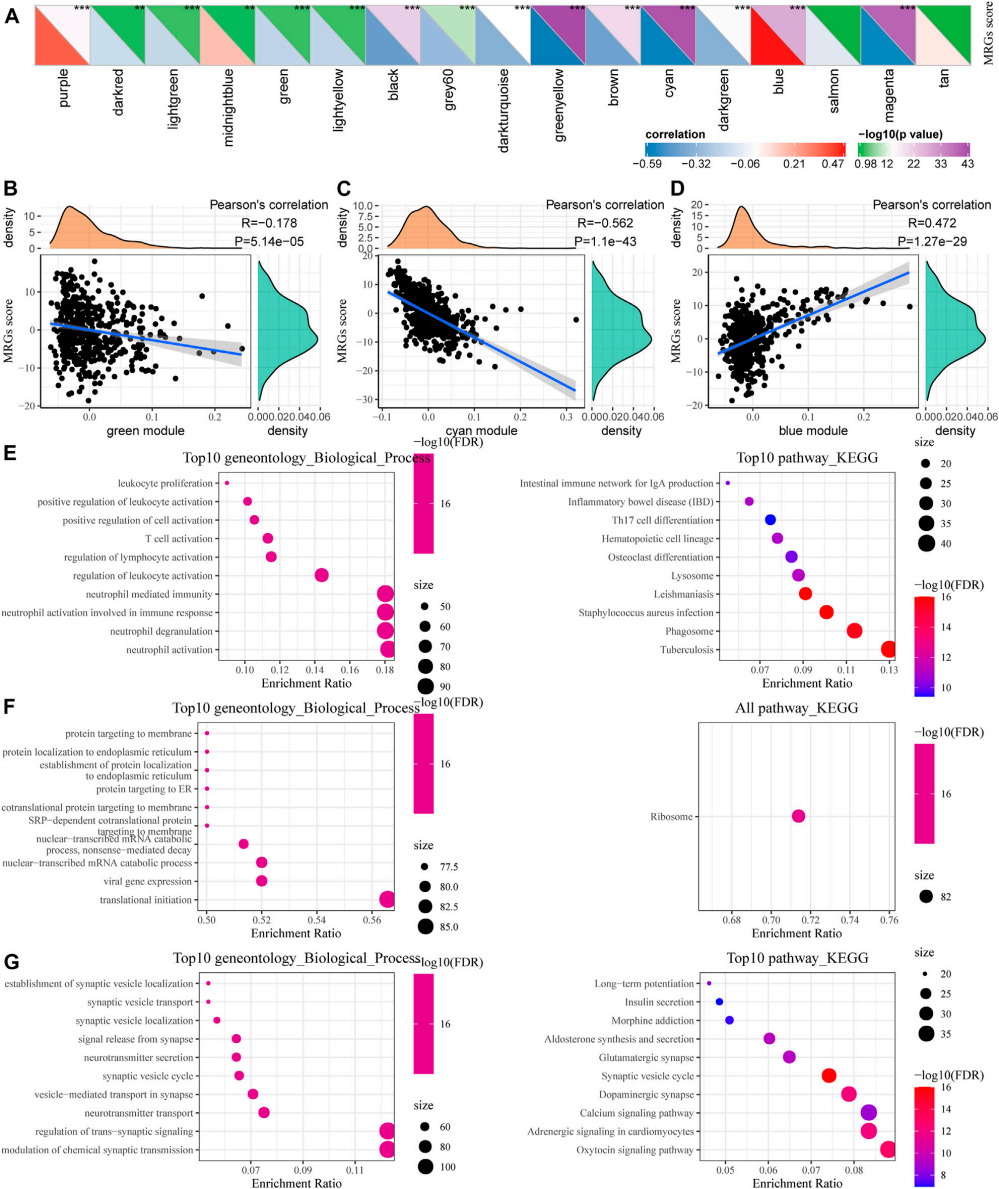
Functional enrichment analysis of metabolic co-expression gene module. **(A)** Correlation analysis between module feature vector and metabolic subtype feature index. **(B)** The correlation between the feature vector of the green module and the feature index of metabolic subtypes. **(C)** Correlation between the cyan module and the feature index of metabolic subtypes. **(D)** Correlation between the blue module feature vector and the metabolic subtype feature index. **(E–G)** Functional enrichment analysis results for green, cyan, and blue modules.

We calculated the correlation between the feature vectors of the 17 modules (excluding the Grey module) and the metabolic subtype feature index, as shown in Figure 7A. The Green and Cyan modules had significant negative correlation with the metabolic subtype feature score (Figures 7B,C). The blue module was significantly positively correlated with the metabolic subtype characteristic score (Figure 7D). We screened the genes of the three modules for functional enrichment, with the following results. As shown in Figures 7E–G, our green module is related to leukocyte proliferation, positive regulation of leukocyte activation, T cell activation, and other immune processes. The KEGG pathway was enriched in the intestinal immune network for IgA production and Th17 cell differentiation. The Cyan module was significantly enriched to protein targeting to membrane, protein localization to endoplasmic reticulum, and other processes (Figure 7F). Furthermore, the results of significant enrichment in the blue module are shown in Figure 7G.

### Hub Gene Selection and Prognosis Analysis

**FIGURE 8:**
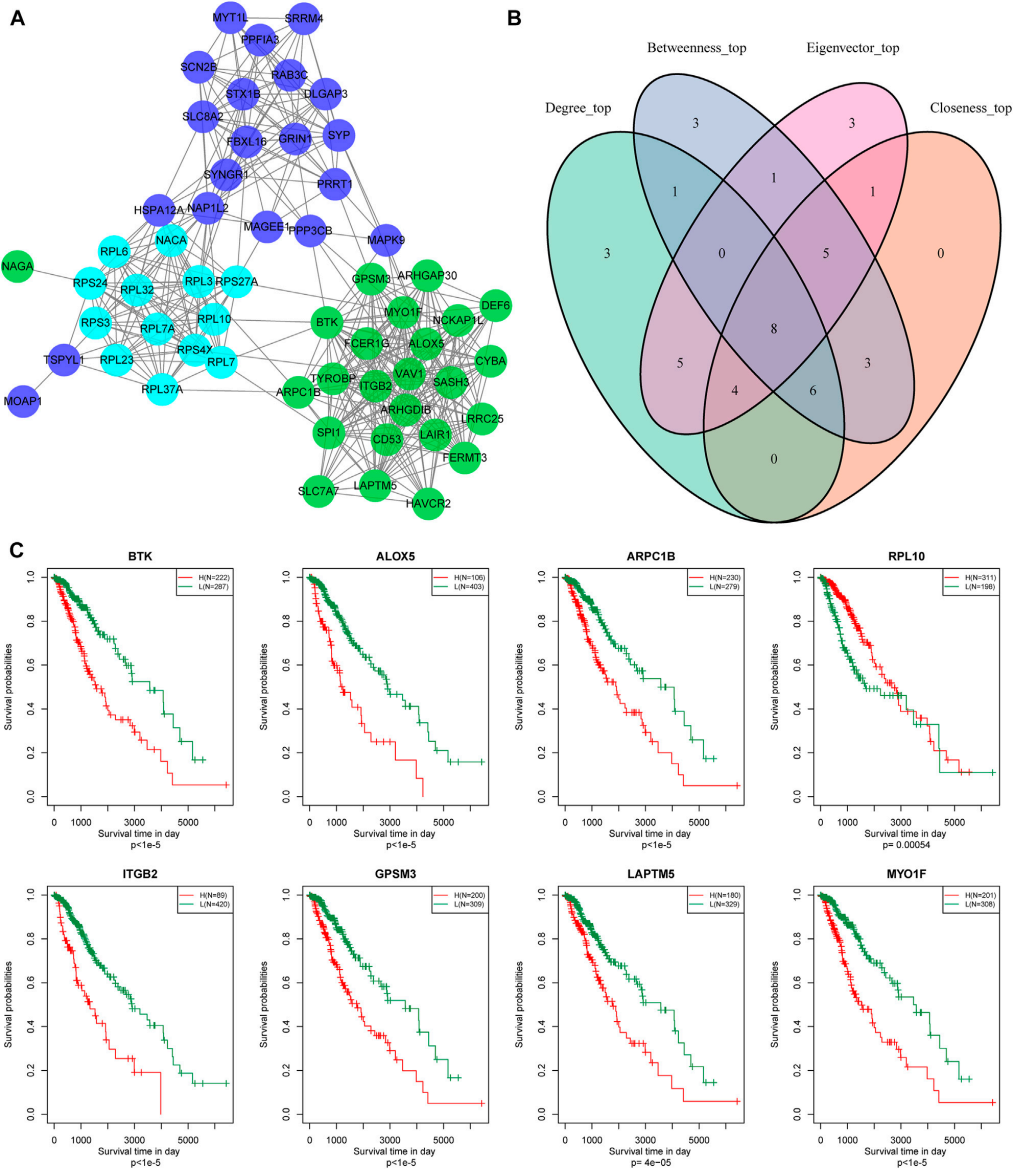
Hub genes identification of metabolic co-expressed gene module. **(A)** The protein interaction network between the key genes of the modules; the different colors of the network nodes indicate different modules. **(B)** Venn diagram of key genes. **(C)** Kaplan-Meier prognostic curve of marker genes related to metabolic subtype feature score.

In addition, we identified 24, 13, and 21 key genes in the green, Cyan, and Blue modules, respectively, with correlation coefficients greater than 0.9 and significant association with prognosis (Figure 8A). We then used a Venn diagram to identify the intersection of a total of eight key genes: BTK, ALOX5, ARPC1B, REL-10, ITGB2, GPSM3, LAPTM5, and MYO1F (Figure 8B).

According to gene expression levels, patients were divided into a high-expression group and low-expression group. Kaplan-Meier prognostic analysis revealed that high expression levels of these eight genes were correlated with poor prognosis (Figure 8C). In conclusion, these eight genes are potential markers associated with the metabolic subtype characteristics score.

## Discussions

LGG is the most commonly diagnosed brain tumor (Merchant et al., 2009). Metabolism-associated genes are reported to play an important role in the tumor microenvironment and tumor genotype construction (Bi et al., 2020). A series of studies have demonstrated that IDH mutation, 1p19q mutation, and MGMT methylation status are the most common molecular mutations with noteworthy (Jenkins et al., 2006; Eckel-Passow et al., 2015). These three mutations occur earlier than glioma formation, and are widely expressed in glioma. They have vital early diagnosis and long-term prognosis prediction value in clinical treatment. In this study, we applied bioinformation analysis to identify the distribution of metabolism-associated genes, and classified these genes into three different clusters with different molecular characteristics. In addition, we analyzed the clinical features of the three clusters. The results demonstrated that the MC3 cluster predicted poor prognosis, combined with IDH mutation, 1p19q mutation, and high MGMT promoter methylation. These metabolic gene-based classifications are linked to specific characteristics, which are well characterized and have overlapping as well as distinct functions. This classification is of direct clinical importance and contributes to improved outcomes in patients with LGG. We have demonstrated that the metabolic profiles of the glioma cell lines are significantly associated with their malignant features. Thus, metabolic prognostic risk signatures that combine the expression of multiple metabolism-related genes will be helpful for the diagnosis, treatment, and prognosis of LGG. The present study identified eight hub signature genes such as BTK, ALOX5, ARPC1B, REL-10, ITGB2, GPSM3, LAPTM5, and MYO1F. These genes were metabolic signatures associated genes. BTK was a non-receptor kinase belonging to the Tec family of kinases which played a vital role in the proliferation and survival of malignant activities (Ahn and Brown, 2021). ARPC1B played an essential role in the maintenance and assembly of the ARP2/3 complex and could function in multiple cellular activities, such as cell migration, progression, and DNA repair (Sprenkeler et al., 2021). ALOX5 and its metabolite 5-hydroxyeicosatetraenoic acid (5-HETE) were involved in tumorigenesis, development, and metastasis (Weigert et al., 2018). Besides, ITGB2 was one subunit of the β2 integrins, which were heterodimeric surface receptors expressed by leukocytes. Additionally, ITGB2 was involved in the development, metastasis, and invasion of various tumors (Xu et al., 2021). It was reported that GPSM3 could act as an NLRP3-interacting protein and a negative regulator of IL-1β production triggered by NLRP3-dependent inflammasome activators (Giguère et al., 2014). LAPTM5, a protein, is preferentially expressed in immune cells (Berberich et al., 2020), and could interact with the Nedd4 family of ubiquitin ligases, which played an essential role in multiple tumor initiation and progression. Finally, MYO1F functioned as an unconventional myosin (Diquigiovanni et al., 2018; Sun et al., 2021) and promoted the expression of critical genes for antifungal innate immune signaling and proinflammatory responses.

Human gliomas are molecularly heterogeneous tumors (Mahlokozera et al., 2018). Tumor heterogeneity in glioma presents a formidable obstacle to personalized therapies (Kondratova et al., 2019). Oncogenic driver mutations may influence tumor initiation and LGG burden, as well as progression to lethal high-grade gliomas (Vitucci et al., 2017). By conducting metabolic profiling on LGGs, we discovered that the metabolic subtypes are associated with particular characteristics, mutation signatures, and intra-tumor heterogeneity. In clinical prognosis, MC1 had the shortest overall survival time compared with the other subgroups. We suspected that the poor prognosis linked with MC1 was mainly due to high EGFR mutations, broad copy number amplification, and high frequency of deletion. Epigenetically regulated mRNAsi, a stemness index, shows a negative correlation with tumor pathology and clinical features (Malta et al., 2018) which mainly results from a high frequency of IDH1/2 mutations and resulting DNA hypermethylation. Consistent with previous research, MC1 had a higher proliferation index and a low mRNAsi index. The MC3 subgroup had a better prognosis mainly because MC3 exhibited high IDH mutation and 1p19q combination deletion. TP53, IDH1, ATRX, and EGFR genes are widely mutated in tumors (Lhomond et al., 2018). The mutation signature results demonstrated that MC3 had high IDH1 mutations. The heterogeneric studies showed that MC3 had the lowest proliferation index, tumor purity ploidy, and intratumor heterogeneity, but a high mRNAsi index.

Growing evidence has demonstrated that metabolic alterations have a profound impact on the fate of immune cells (Wang et al., 2021). Cellular metabolism is involved in immune cell composition and immune response (Tomas et al., 2018). In immune cells, infiltration is typically correlated with favorable prognosis and immunotherapy response (Robertson et al., 2017). In our study, inhibitory immune cells (M2 macrophages) were highly infiltrated in the MC1 subgroup. In addition, MC1 contained the highest frequency of the C4 subtype previously reported in the immune cell landscape (Thorsson et al., 2019). Immune checkpoint blockade has led to great achievements in cancer therapy (Chen et al., 2016), but the response to immune therapy is limited (Chen et al., 2016). The MC1 subgroup was sensitive to anti-PD1 therapy, which may pave the way for LGG-associated immunotherapies.

In conclusion, we established a stable metabolic signature classification model and identified eight potential metabolic biomarkers for LGG prognosis and progression.

## Data Availability

The datasets presented in this study can be found in online repositories. The names of the repository/repositories and accession number(s) can be found in the article/Supplementary Material.
