# Supplementary figures and images for: Metabolic Signature-Based Subtypes May Pave Novel Ways for Low-Grade Glioma Prognosis and Therapy
Source: Front Cell Dev Biol. 2021 Nov 23;9:755776. doi: 10.3389/fcell.2021.755776 (PMC8650219; doi:10.3389/fcell.2021.755776)

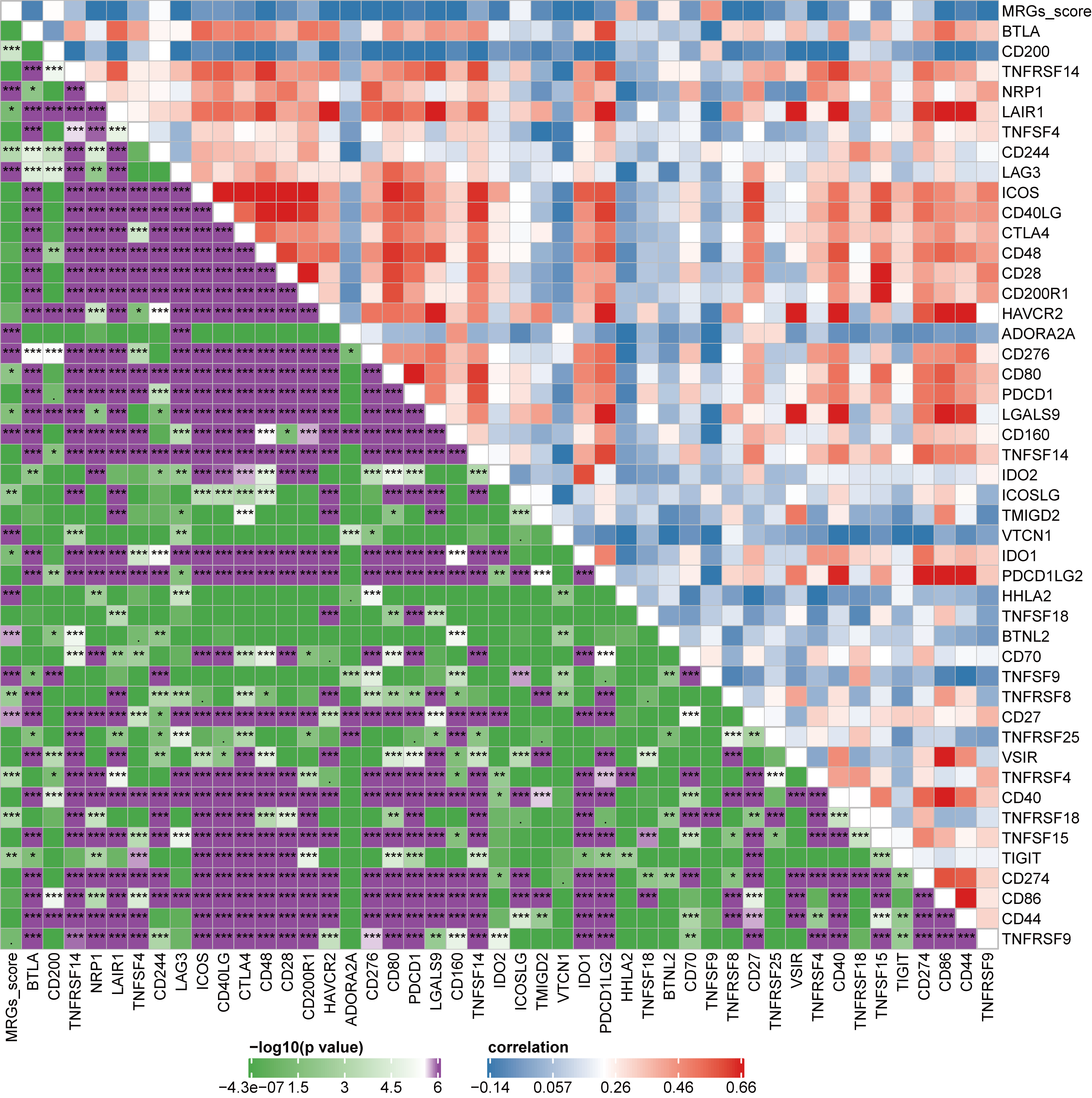

Supplement: Supplementary file 1 [file Image6.TIF]

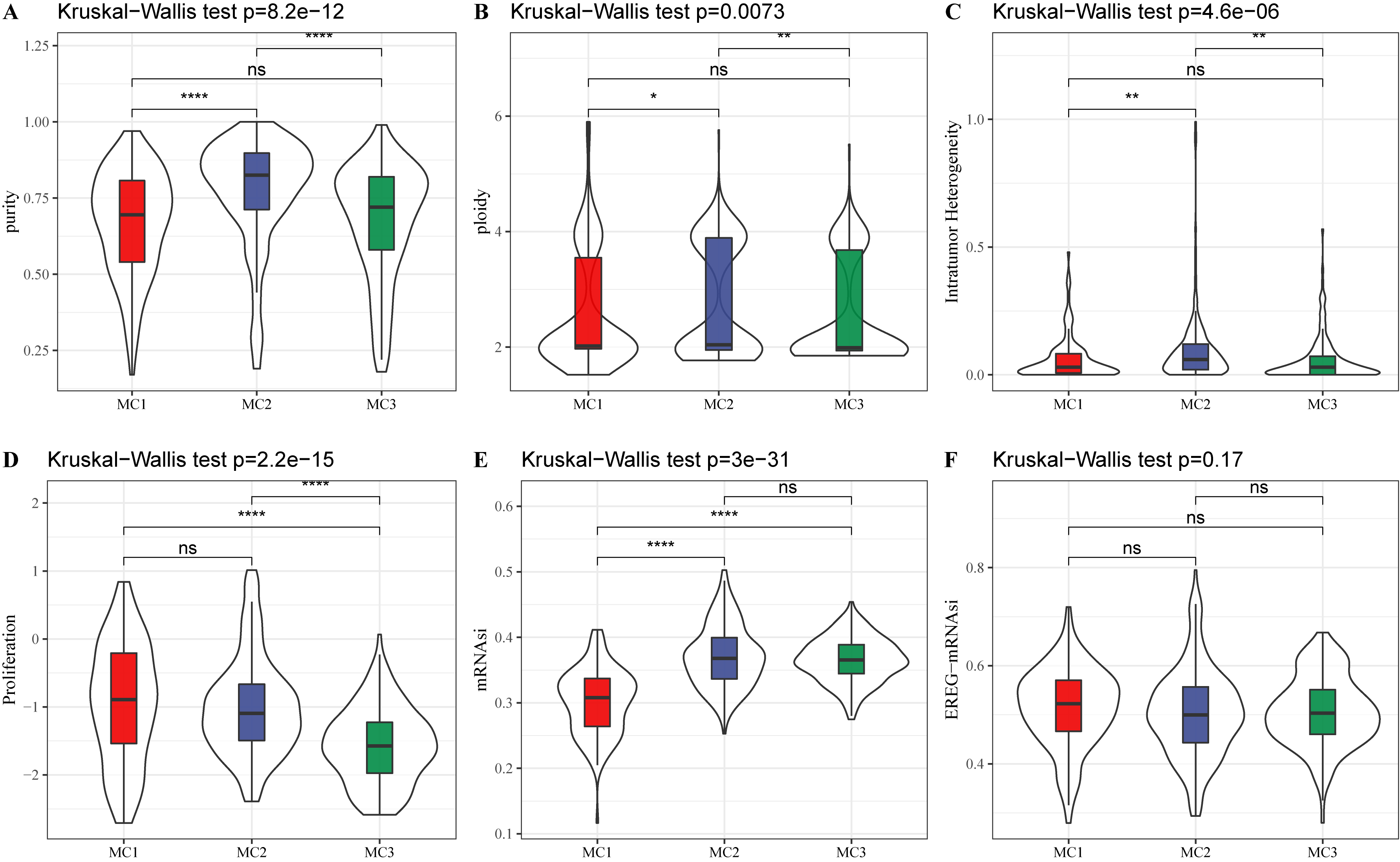

Supplement: Supplementary file 2 [file Image3.TIF]

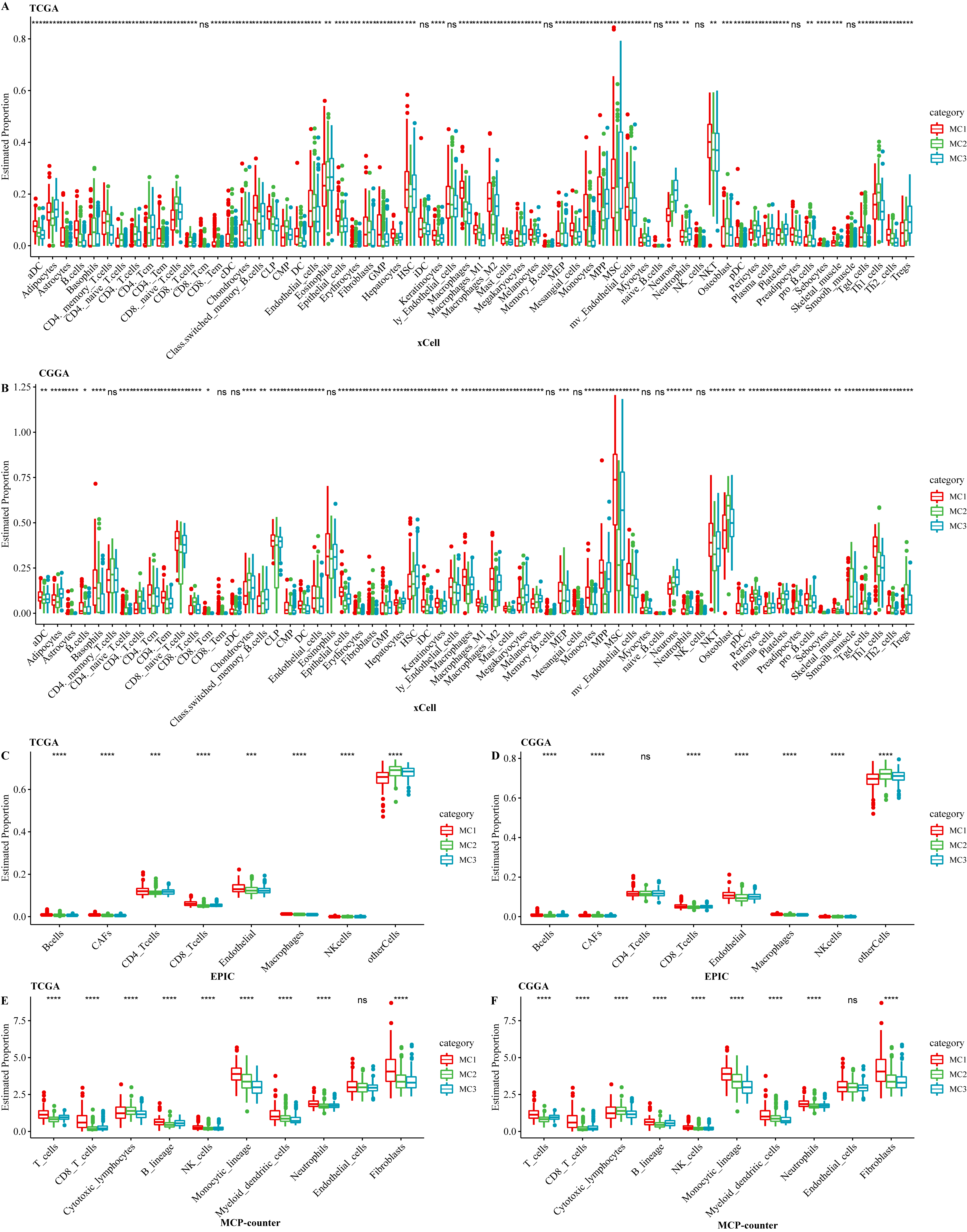

Supplement: Supplementary file 3 [file Image4.TIF]

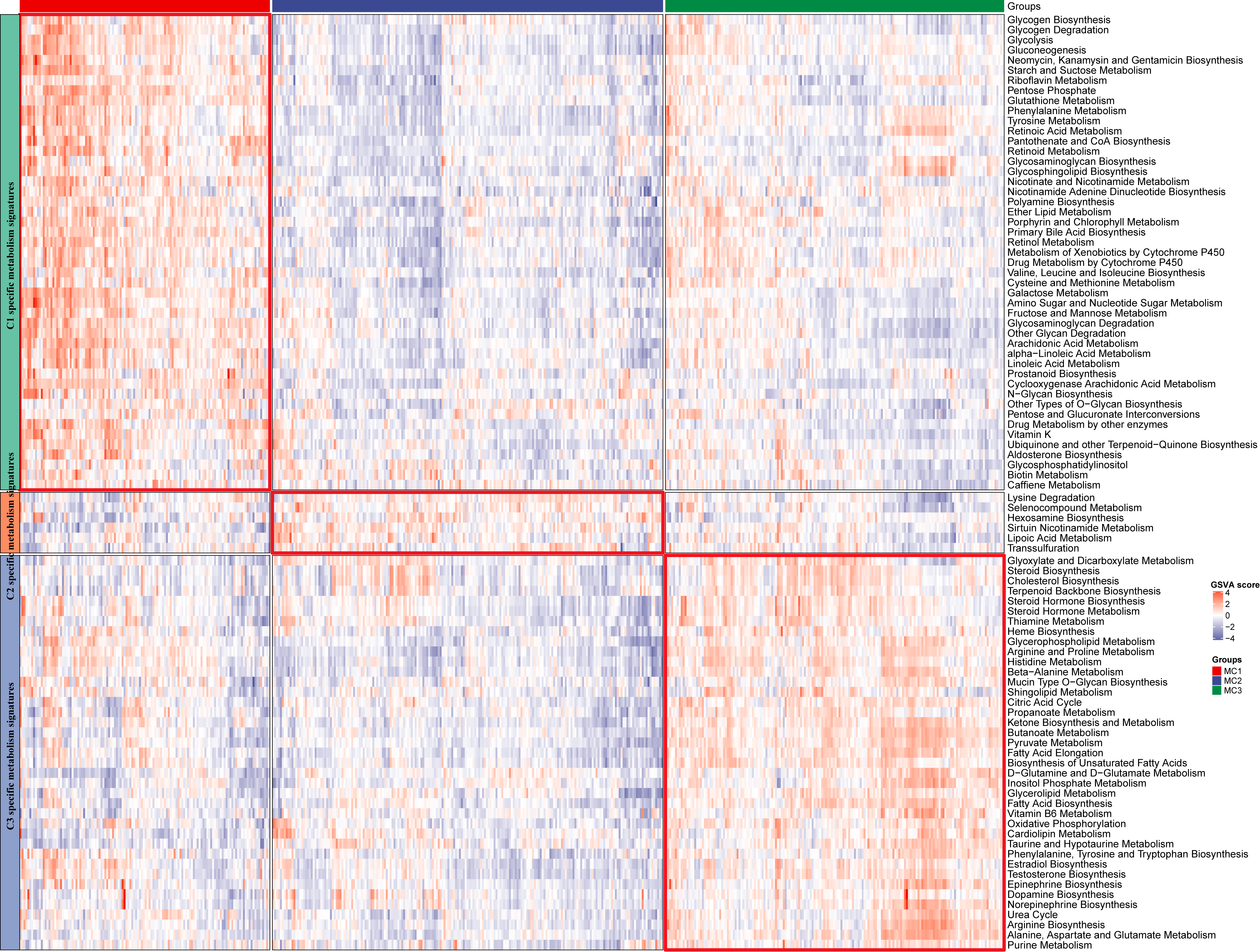

Supplement: Supplementary file 4 [file Image2.TIF]

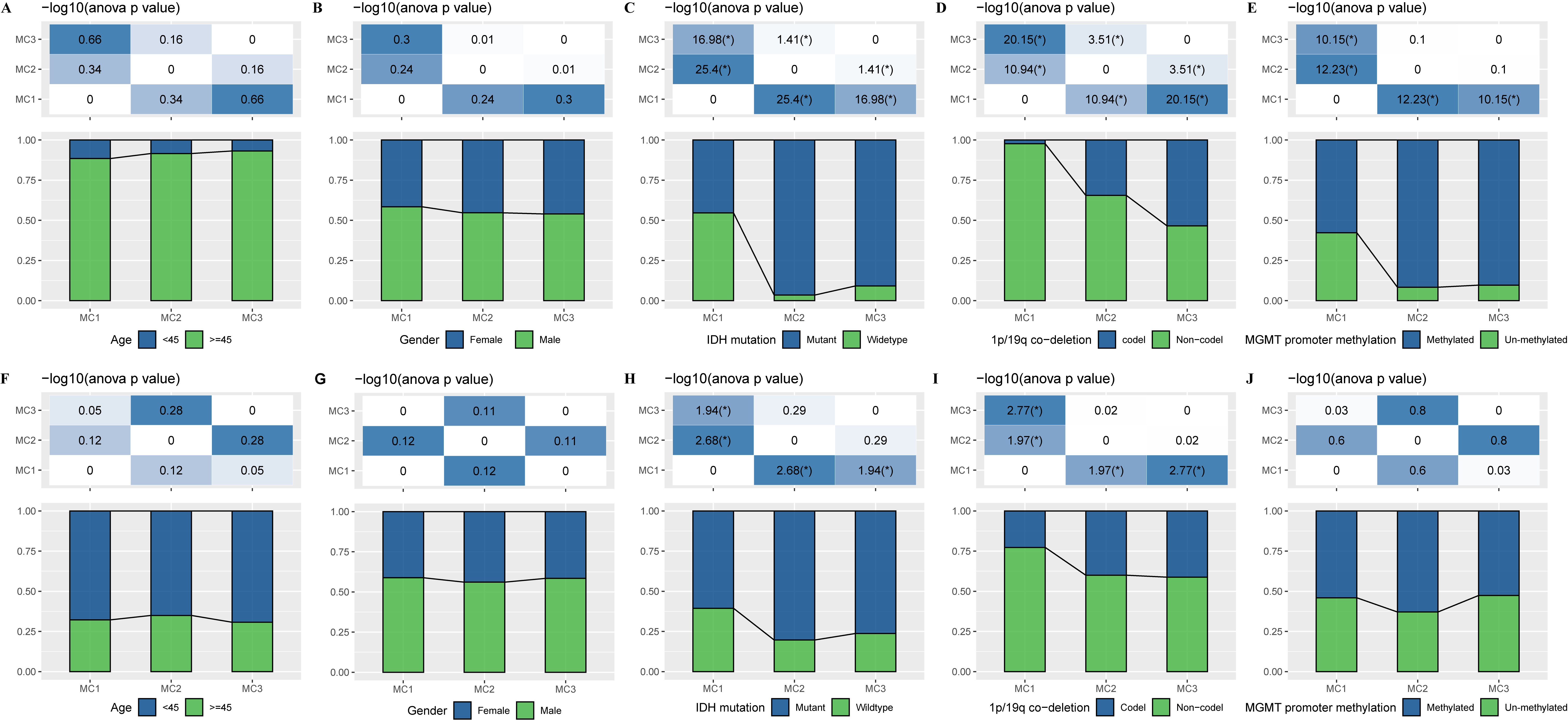

Supplement: Supplementary file 5 [file Image1.TIF]

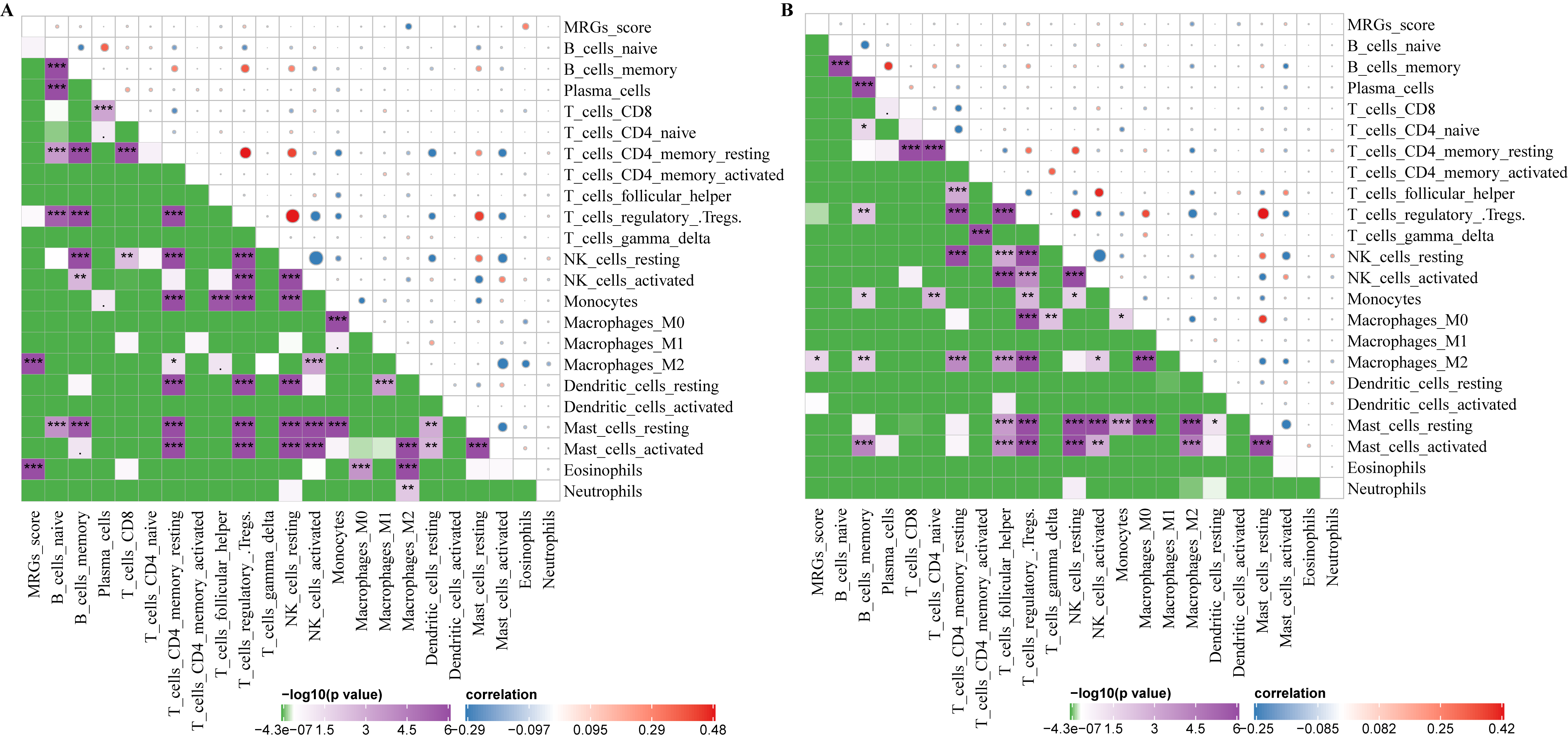

Supplement: Supplementary file 6 [file Image5.TIF]
